# Preference for Boys, Family Size, and Educational Attainment in India

**DOI:** 10.1007/s13524-017-0575-1

**Published:** 2017-05-08

**Authors:** Adriana D. Kugler, Santosh Kumar

**Affiliations:** 1 McCourt School of Public Policy, Georgetown University, 311 Old North Building, 37th and O Streets, NW, Washington, DC 20057 USA; 2 National Bureau of Economic Research (NBER), 1050 Massachusetts Avenue, Cambridge, MA 02138 USA; 3 Centre for Economic Policy Research (CEPR), 33 Great Sutton Street, London, EC1V 0DX UK; 4 Department of Economics, Centre for Research & Analysis of Migration (CReAM), Drayton House, 30 Gordon Street, London, WC1H 0AX UK; 5 Institute of Labor Economics (IZA), Schaumburg-Lippe-Strasse 5-9, 53113 Bonn, Germany; 6 Department of Economics and International Business, Sam Houston State University, Huntsville, TX 77341-2118 USA

**Keywords:** Quantity-quality trade-off, Education, Family size, India

## Abstract

Using data from nationally representative household surveys, we test whether Indian parents make trade-offs between the number of children and investments in education. To address the endogeneity due to the joint determination of quantity and quality of children, we instrument family size with the gender of the first child, which is plausibly random. Given a strong son preference in India, parents tend to have more children if the firstborn is a girl. Our instrumental variable results show that children from larger families have lower educational attainment and are less likely to be enrolled in school, with larger effects for rural, poorer, and low-caste families as well as for families with illiterate mothers.

**Electronic supplementary material:**

The online version of this article (doi:10.1007/s13524-017-0575-1) contains supplementary material, which is available to authorized users.

## Introduction

High population growth has long been considered a potential deterrent for economic growth and development. By contrast, human capital accumulation is considered one of the main determinants of income growth. At the household level, family size and human capital are also negatively correlated: a larger family has fewer resources to devote to each child’s education. That is, in making child rearing decisions, resource-constrained households may face a quantity-quality (Q-Q) trade-off, a concept originally developed by Becker and Lewis (1973).

In this study, we test the empirical validity of the child Q-Q trade-off in India. Q-Q trade-offs are likely to be stronger in a country like India, where households are more likely to face resource constraints. We exploit the cultural phenomenon of son preference in India as a natural experiment to examine the causal effect of family size on parental investments in their children. The Indian context is important in its own right for studies of low human capital investments in one of the most populous countries in the world, with more than 1.2 billion people. According to the 2013/14 Education for All Global Monitoring Report, India has the highest population of illiterate adults, at 287 million, amounting to 37 % of the global total (UNESCO 2015). The national dropout rate at the primary level was 4.3 % in 2014–2015, and it was even higher at the secondary level, at 17.8 %. The overall learning level among Indian school students is low; only 50 % of grade V students can read text of grade II (Pratham Education Foundation 2017).

Empirical testing of the Q-Q trade-off is challenging because fertility decisions and investments in children are jointly determined and depend on common factors (Browning 1992; Haveman and Wolfe 1995). Omitted variable bias of this type will tend to exaggerate the negative relation between family size and human capital investments. To address this concern, we employ an instrumental variable (IV) method and use gender of the first child to instrument family size. The social norm of son preference in India means that when a household has a firstborn girl, parents continue to have more children until they have the desired number of boys in the family. Son preference—widely documented in countries such as India, China, and Korea—is deeply rooted in social, economic, and cultural factors (Pande and Astone 2007). Moreover, there is little evidence that households with firstborn girls are different in other ways from those with firstborn boys; this satisfies the exclusion restriction of the instrument.

We use the District Level Household Survey (DLHS) from 2007–2008 to examine the impact of family size on educational achievements in India. The IV results show that in the average family, having an extra child in the family reduces schooling by more than one-quarter of a year and reduces the probability of being enrolled in school or ever attending school by approximately 1 and 2 percentage points, respectively. We also find heterogeneous effects, with larger Q-Q trade-offs for rural, poor, and low-caste households as well as for households with illiterate mothers. The impact of having an extra child in terms of reducing enrollment and attendance roughly doubles, and the impact of having an extra child on years of schooling increases approximately threefold for illiterate and poor mothers, suggesting much larger gains from reducing family size in disadvantaged households.

## Literature Review

Since Becker and Lewis (1973) developed the Q-Q model, a number of studies have tried to quantify the magnitude of the Q-Q trade off. These studies addressed the endogeneity of family size by taking advantage of exogenous variation in policy experiments (e.g., the one-child policy in China), natural occurrences of twin births, and sibling sex composition. The original causal test of the Q-Q trade-off used data from India in the 1980s (Rosenzweig and Wolpin 1980), and there has been renewed attention on this topic in developed and developing countries over the last decade.

The birth of twins is the most commonly used exogenous increase in family size to study the Q-Q trade-off in high-income countries. Black et al. (2005) used twins as an instrument for family size using Norwegian data and found no evidence that family size affects educational attainment of children, after controlling for birth order. Similarly, Angrist et al. (2010) used multiple births and same-sex siblings in families with two or more children as instruments for family size in Israel. They also failed to find a significant relation between family size and schooling and employment. De Haan (2010) found no significant effect of family size on the educational attainment of the oldest child in the United States or the Netherlands. However, a few studies in developed countries did find evidence of a Q-Q trade-off (Caceres-Delpiano 2006; Conley and Glauber 2006; Goux and Maurin 2005).

Small or no effects of family size on human capital investments in developed countries may be due to the presence of well-functioning public education systems, which may substitute for private education and may still allow parents to provide a good education (Li et al. 2008). By contrast, child labor practices and the absence of good public education may make this trade-off more pronounced in developing countries.

Rosenzweig and Wolpin (1980) were the first to exploit twins as an exogenous shock to family size, finding a weak negative effect on educational attainment for nontwin children in India. The study, however, was based on a small nonrepresentative sample of 1,633 households that included only 25 households with twins.

In recent years, there has been renewed attention to the Q-Q literature in the context of developing countries. Evidence on the Q-Q trade-off in China is mixed. Using data from the 1 % sample of the 1990 Chinese Census, Li et al. (2008) relied on twin births as an instrument and found that larger family size reduces a child’s education even after birth order is controlled for, especially in rural China. Using twins as an exogenous shock to family size, Rosenzweig and Zhang (2009) showed that having an extra child significantly decreases educational attainment. However, they argued that the use of twins as an instrument generates upward biases because of differences in birth weight between twins and nontwins, which changes parental behavior and overall resource allocation within the household.

Studies for other developing countries that relied mainly on the twinning experiment have tended to show either small or no effects. Using twinning as an instrument, Ponczek and Souzay (2012) also reported negative effects on educational outcomes in Brazil. Additionally, Glick et al. (2007) used twinning at first birth and found that unplanned fertility increases the nutritional status and school enrollment of later-born children in Romania. Instrumenting family size by the commuting distance to the nearest family planning center, Dang and Rogers (2016) showed that larger family size reduces investments on schooling in Vietnam.

To our knowledge, only a handful of studies have used son preference as an instrument to study Q-Q trade-offs in Asian countries (Lee 2008; Sarin 2004). Sarin (2004) found no empirical relationship between family size and weight-to-height ratio among children in India. Lee (2008) also instrumented family size by gender of the first child to examine the effect of family size on education in South Korea. Using parity progression and Weibull hazard models of fertility timing, Lee (2008) first showed that “first girl” can be a good instrument for family size in South Korea, where strong preferences for sons and small families are social norms.^1^ He ruled out that sex-selective abortions and postnatal son preferences might invalidate the instrument. His study used parents’ monetary investment in children’s education as a measure of child quality instead of schooling outcomes^2^ and showed that the elasticity of per child investment with respect to family size ranged from −0.29 to −0.37 and that this trade-off became stronger with increasing numbers of children in the family.

Our study makes several important contributions to the literature. First, our study contributes to the child Q-Q trade-off literature in India, where son preference and larger family size are norms. Second, the data allow us to use gender of the first child as an instrument for family size, combined with good measures of child quality. This feature is important because most studies have relied on twinning experiments, and now there is enough evidence that twins are differentially and poorly endowed at birth (Rosenzweig and Zhang 2009). Third, although several studies have focused on China and other regions of the developing world, ours is among a handful of studies to estimate the impact of family size on educational outcomes in India. Not only is India host to 17 % of the world’s population and important in its own right, but its lack of quality educational infrastructure is likely to exacerbate the severity of the Q-Q trade-off. Finally, we examine nationally representative samples of the Indian population, which has not been always the case for Q-Q trade-off studies in other developing countries.

## Empirical Framework

We first estimate the effect of family size on children’s educational outcomes using the following ordinary least squares (OLS) model:

1 $$ {Y}_{chd}={\upbeta}_0+{\upbeta}_1{FamilySize}_{hd}+{\upbeta}_2{\mathbf{X}}_{1_{chd}}+{\upbeta}_3{\mathbf{X}}_{2_{hd}}+{\upmu}_d+{\upvarepsilon}_{chd}, $$

where *Y*_*chd*_ is the educational outcome of child *c* in household *h* residing in district *d*. The educational outcomes of the child are the probability of ever attending school, the probability of being currently enrolled in school, and years of schooling. *FamilySize*_*hd*_ is the number of surviving children under 21 years of age residing in the household at the time of the survey.^3^ The DLHS data set contains neither information about children who have moved or married out nor information about total ever-born children in the family, so we are constrained to use number of surviving and resident children as the measure of family size.^4^ ${\mathbf{X}}_{1_{chd}}$ is a vector of child-level covariates (age, age squared, gender, and birth order), ${\mathbf{X}}_{2_{hd}}$ is a vector of parent/household-level covariates (religion, caste, wealth index, mother’s age, father’s age, mother’s education, father’s education, and a rural dummy variable), and ε_*chd*_ is an error term. μ_*d*_ are district fixed effects that adjust for time-invariant characteristics of the districts.

A negative coefficient of β_1_ would capture the Q-Q trade-off. β_1_ will, however, provide the causal impact of family size on child quality only if family size is exogenously determined. On the other hand, if decisions about fertility and investments in children are determined simultaneously, the OLS estimate of β_1_ in Eq. (1) is subject to endogeneity bias and is unlikely to capture the causal effect of family size on child quality. OLS estimates may be downwardly or upwardly biased depending on the source of the endogeneity. For example, in a country like India, wealthier households may have fewer children and may invest more in their children’s schooling, thus generating an upward bias in the Q-Q trade off. However, highly committed parents may have more children and may invest more in their children’s education, thus generating a downward bias.

Therefore, we rely on the IV method and estimate a two-stage least squares (2SLS) model to capture only exogeneous variation in family size. The key is to identify a variable that predicts *FamilySize* but is uncorrelated with the error term in Eq. (1). We use an indicator for a firstborn girl (FBG) as an instrument and estimate the following 2SLS model:

2 $$ {FamilySize}_{hd}={\upalpha}_0+{\upalpha}_1{FBG}_{hd}+{\upalpha}_2{\mathbf{X}}_{1_{chd}}+{\upalpha}_3{\mathbf{X}}_{2_{hd}}+{\upmu}_d+{u}_{chd} $$

3 $$ {Y}_{chd}={\uppi}_0+{\uppi}_1 Fam\widehat{ilySi}{ze}_{hd}+{\uppi}_2{\mathbf{X}}_{1 chd}+{\uppi}_3{\mathbf{X}}_{2 hd}+{\upmu}_d+{v}_{chd} $$

where *FBG*_*hd*_ is a dummy variable that equals 1 if the firstborn is a girl, and 0 otherwise. This approach is similar in spirit to that of Lee (2008) and Angrist and Evans (1998), who used gender of the firstborn child and first two children, respectively, as instruments for family size. Standard errors are clustered at the district level.

In the 2SLS framework, Eq. (2) is the first-stage regression, and Eq. (3) is the second-stage regression. The second stage regresses the measures of child quality on the predicted value of family size from Eq. (2) and other exogenous variables. We also estimate the 2SLS regressions for a number of subgroups, including different castes, households with different levels of wealth, and households with different levels of educational attainment of the mother and for urban and rural subsamples, separately.

A key condition for the gender of the first child to be a valid instrument is for family size to be highly correlated with the gender of the first child—that is, Corr(*FBG*, *FamilySize*) ≠ 0. In India, there is a long-standing social and cultural norm of son preference for several reasons (Pande and Astone 2007). First, only sons are allowed to carry forward the family legacy and name. More importantly, because India is a patriarchical society, sons inherit the family’s patrimony. Second, parents prefer male children because sons are expected to provide financial support and care for their parents in old age. In addition, because men are more likely to enter the labor force and earn higher wages, these gender gaps in the labor market further contribute to a family’s preference for boys. In Indian tradition, daughters are married out and become part of another family. Because parents provide a dowry when daughters marry, families prefer to have boys so they can receive a dowry when their sons marry. In this type of patrilineal familial system, if the firstborn is a girl, parents are likely to continue having children until a son is born. In the upcoming section, Effects of Family Size on Educational Attainment, we test for this by estimating the first-stage relationship in Eq. (2).

The second key assumption behind this identification strategy is that the gender of the firstborn is uncorrelated with educational outcomes other than through family size—that is, Corr(*FamilySize*, *v* ) = 0. Because gender of the first child is determined by nature, this is considered a random event that is uncorrelated with educational attainment. However, if parents have any control over births and make decisions about births depending on sex, the sex of the first birth will not be random. Therefore, sex-selective abortions may invalidate the instrument because access to ultrasound technologies and abortion services allows parents to choose the sex of their children. However, sex-selective abortions are not as big a concern given that the Pre-natal Diagnostic Techniques Act passed in India in 1996 made fetal-sex determination illegal. In addition, many previous studies have shown that parents in India do not use sex-selective abortions for firstborns but only for subsequent births. These studies found that the sex ratio at first birth lies within the biologically normal range of 1.03–1.07 (Bhalotra and Cochrane 2010; Jha et al. 2011; Portner 2015; Rosenblum 2013a).^5^

Using the same data as ours, Rosenblum (2013a) reported a lack of sex-selection abortion at first parity and showed that 36 % of women reported induced abortions at the second and third parities. Additionally, using the first two rounds of the National Family and Health Survey (NFHS), Retherford and Roy (2003) reported little or no evidence of sex selection at the first birth. Sociological studies have also provided evidence that parents have a strong preference for sons only after the first birth (Patel 2007). Taken together, these studies provide credible evidence that sex of the firstborn is indeed exogenous and random. To further confirm the exogeneity of the instrument, we explore whether the instrument, *FBG*, is correlated with observable characteristics of the household to gauge whether the sex of the firstborn can also be assumed to be uncorrelated with unobservable characteristics. We also test for sex-selective abortion with our data to see whether the firstborn is more likely to be male.

## Data Description

We use data from the third round of the Indian District Level Household Survey (DLHS), collected in 2007–2008, and the first round of the NFHS (1992–1993) for our analysis. The DLHS sample is representative at the district level, which is the lowest tier of administration and policy-making in India. The DLHS covers 601 districts and on average draws a random sample of 1,000–1,500 households from each district (International Institute for Population Sciences 2010).

Our analysis uses the household questionnaire of the DLHS, which collected information on assets and socioeconomic characteristics, including the following information for each household member: age, gender, schooling attendance, and years of completed schooling. We identify individuals who are labeled sons/daughters and estimate the family size by counting the number of sons/daughters in the household at the time of the survey; we then merge these data with the parents’ information.

We restrict the sample in the following ways. First, we restrict the sample to individuals who are either parents (head of the household and spouse) or sons/daughters of the head of the household.^6^ Second, we restrict the sample to households with two or more births so that we can use the gender of the first child as an instrument. Third, we restrict the sample to school-aged children who are aged 5–20. We use 5 as the lower age bound because the survey collects education information only for individuals who are 5 years or older. In India, primary school (grades 1 to 5) begins at age 5 or 6 and ends at age 10 or 11, and high school is typically completed by age 18. However, given that completion of either primary or secondary schooling might be delayed because of deferred enrollment or grade repetition, we include children until age 20. We exclude mothers over age 35 to minimize the possibility that adult children may have already left the household, especially older girls who are less likely to be observed in the data because of marriage. Finally, we exclude households with missing or unreliable information on any of the variables used in the analysis. Less than 2 % of the sample were dropped due to missing information, yielding an analytical sample of 393,510 children.

We use three measures of educational attainment: (1) an indicator of whether the person ever attended school; (2) an indicator of whether the person is currently enrolled in school; and (3) years of schooling. We control for the following child-level covariates: age, age squared, gender, and birth order. In addition to age and gender, birth order has been found to be correlated with educational attainment in India (Kumar 2016). We additionally control for the following parental-level characteristics: caste, religion, a rural indicator, an asset-based standard of living index, mother’s age, father’s age, mother’s education, and father’s education. We divide caste into three groups: (1) scheduled caste and scheduled tribe are combined to constitute the low-caste category (a group that is socially segregated and disadvantaged); (2) other backward classes (officially identified as socially and educationally backward) are considered as the middle-caste category; and (3) the upper caste (comprising Brahmins and other higher castes who are privileged) are classified as high caste. Religion is included as a Hindu dummy variable. The rural indicator is constructed using the DLHS definition of rural and urban areas, which is based on population size, share of the population engaged in agrigultural/nonagricultural activities, and population density.^7^ The DLHS data do not contain information on individual or household incomes. The survey does ask, however, a multitude of questions about the ownership of assets, including ownership of a car, television, real state property, and other assets. The DLHS uses ownership of assets to create a standard of living index with three categories: low, middle, and high.^8^

Table 1 reports the summary statistics of individual and household characteristics for the estimation sample. The average age of children in the sample is 9.6 years, and the average number of years of schooling is 3.08. Approximately 49 % of firstborn children are female. Fathers are older than mothers: the average age is 31 years for mothers and 36 for fathers. The average years of schooling for mothers and fathers are 3 and 5.5 years, respectively. The average family size is 3.54. Approximately 82 % of children live in rural areas. In terms of caste, 41 %, 39 %, and 20 % of the children come from a low-, middle-, and high-caste household, respectively. Finally, 49 %, 39 %, and 12 % of children have the lowest, middle, and highest standard of living index, respectively.

**Table 1:** Descriptive statistics of the sample

	All	Firstborn Girl	Firstborn Boy
Children’s Age	9.60	9.41	9.79
	(3.45)	(3.34)	(3.55)
Firstborn Girl	0.49		
	(0.49)		
Ever Attended School	0.90	0.89	0.91
	(0.30)	(0.31)	(0.29)
Currently Enrolled	0.95	0.95	0.95
	(0.21)	(0.21)	(0.22)
Years of Schooling	3.08	2.94	3.22
	(2.92)	(2.85)	(2.98)
Mother’s Age	30.94	30.88	31.00
	(3.36)	(3.34)	(3.37)
Father’s Age	36.48	36.42	36.54
	(4.81)	(4.79)	(4.82)
Mother’s Years of Schooling	2.99	3.05	2.93
	(4.06)	(4.09)	(4.03)
Father’s Years of Schooling	5.48	5.56	5.40
	(4.74)	(4.76)	(4.72)
Family Size	3.54	3.70	3.40
	(1.33)	(1.33)	(1.31)
Rural	0.82	0.81	0.82
	(0.39)	(0.39)	(0.39)
Low Caste	0.41	0.41	0.41
	(0.49)	(0.49)	(0.49)
Middle Caste	0.39	0.39	0.39
	(0.49)	(0.49)	(0.49)
High Caste	0.20	0.20	0.20
	(0.40)	(0.40)	(0.40)
Low Wealth	0.49	0.48	0.49
	(0.50)	(0.50)	(0.50)
Medium Wealth	0.39	0.40	0.39
	(0.49)	(0.49)	(0.49)
High Wealth	0.12	0.13	0.12
	(0.33)	(0.33)	(0.32)
Number of Observations	393,510	193,263	200,247
Number of Districts	601		

*Notes:* Standard deviations are shown in parentheses. All sampled children were 5–20 years old at the time of survey (2007–2008). Low caste is scheduled caste (SC) and scheduled tribe (ST) households, and the middle caste is other backward caste (OBC). The analytical sample is restricted to mothers aged 20–35.

### Sex-Selective Abortions and Exogeneity of the Instrument

As shown in Table 1, 49 % of firstborns are female, indicating that the sex ratio at first birth is in the biological range. Table 2 reports the results of linear probability and probit models predicting the likelihood that the firstborn is a girl on the characteristics reported in Table 1 to investigate whether the instrument is likely to be exogeneous. Results in the first two columns show that the explanatory variables, except for mother’s age, are statistically insignificant, which provides additional evidence that the gender of the firstborn is unrelated to observable characteristics and is likely exogenous.

**Table 2:** Regression of firstborn girl on household characteristics

	Dependent Variable: Firstborn Girl		Having a Girl Child
	LPM	Probit	LPM
	(1)	(2)	(3)
Birth Order (1st)	––	––	0.023**
			(0.002)
Rural	−0.003	−0.008	0.004
	(0.004)	(0.011)	(0.003)
Low Wealth	−0.002	−0.004	−0.003
	(0.007)	(0.017)	(0.004)
Medium Wealth	−0.004	−0.009	−0.006^†^
	(0.005)	(0.013)	(0.003)
Hindu	0.006	0.016	0.004^†^
	(0.00)	(0.011)	(0.003)
Low Caste	0.004	0.009	−0.005*
	(0.004)	(0.011)	(0.003)
Middle Caste	0.002	0.006	−0.002
	(0.004)	(0.010)	(0.002)
Mother’s Years of Schooling	0.002	0.004	0.0003
	(0.001)	(0.003)	(0.0003)
Father’s Years of Schooling	−0.0001	−0.0001	−0.0001
	(0.001)	(0.002)	(0.0002)
Mother’s Age	0.037**	0.093**	0.005
	(0.007)	(0.017)	(0.004)
Father’s Age	0.002	0.004	0.002
	(0.003)	(0.008)	(0.002)
District Fixed Effects	Yes	Yes	Yes

*Notes:* Robust standard errors, clustered by district, are shown in parentheses. LPM is linear probability model. Column 2 reports marginal effects from the probit model. Low caste is scheduled caste (SC) and scheduled tribe (ST) households, and the middle caste is other backward caste (OBC).

^†^ *p* ≤ .10; *p ≤ .05; **p ≤ .01

Because sex-selective abortion is a concern, in column 3 of Table 2, we further explore sex selectivity at first birth by estimating a simple linear probability model of the likelihood of having a girl on birth order, controlling for age, religion, caste, mother’s and father’s education and age, socioeconomic status (SES), and whether they live in a rural or urban area. SES is measured using a standard of living index of the household. The results show that the firstborn is more likely to be a girl or less likely to be a boy compared with higher-order births, even when we control for all other characteristics. If sex-selective abortions were prevalent at first birth, the results would show the opposite sign.

Even though our analysis and previous studies show that self-selective abortions are unlikely to be a problem for first births, one of the advantages of the data that we use in this study is that they cover a period after the legal ban on determination of fetal gender. We argue that the post-ban period will be less susceptible to sex-selective abortions because parents are less likely to know the gender of the fetus compared with the pre-ban period. If this is true, then the policy change regarding the legal ban on abortion should matter for our results, and therefore the Q-Q trade-off should be weaker during the pre-ban period. We explore this by using data from the first round of NFHS collected in 1992–1993. We find no evidence of a Q-Q trade-off in the period before the abortion ban (panel A in Table 8 of the appendix). In addition, the NFHS 1992 data show suggestive evidence of sex-selective abortions. In the NFHS 1992 data, households with a firstborn girl are more likely to be wealthy and educated, suggesting that wealthier households have a higher propensity to engage into sex-selective abortions (results available upon request).

## Effects of Family Size on Educational Attainment

### OLS and 2SLS Impacts of Family Size on Schooling

Table 3 reports the OLS results. Columns 1–3 report results that control only for district fixed effects to account for time-invariant district characteristics. Columns 4–6 report results adding children’s controls, and results reported in columns 7–9 additionally control for parents’ characteristics. These results highlight the importance of controlling for parental characteristics. Adding parental controls in columns 7–9 reduces the coefficient of family size for all three educational outcomes. The coefficient on ever attended school falls from −0.03 to −0.018; the coefficient on years of schooling falls from −0.293 to −0.202; and the coefficient on current enrollment falls from −0.019 to −0.014. These results, thus, imply that children in families with one additional child are 1.8 percentage points less likely to have ever attended school, and the likelihood that they are currently enrolled in school is 1.4 percentage points lower. For years of schooling, the point estimate is −0.2, suggesting that children in families with five or more siblings will end up with one year less of schooling, on average.

**Table 3:** OLS estimates of the effect of family size on education

	Ever Attended School	Years of Schooling	Currently Enrolled	Ever Attended School	Years of Schooling	Currently Enrolled	Ever Attended School	Years of Schooling	Currently Enrolled
	(1)	(2)	(3)	(4)	(5)	(6)	(7)	(8)	(9)
Family Size	−0.024**	−0.005	−0.019**	−0.030**	−0.293**	−0.019**	−0.018**	−0.202**	−0.014**
	(0.001)	(0.008)	(0.001)	(0.001)	(0.007)	(0.0007)	(0.001)	(0.006)	(0.0006)
Child-Level Controls									
Child’s age				0.085**	0.852**	−0.059**	0.080**	0.835**	0.061**
				(0.002)	(0.013)	(0.001)	(0.002)	(0.014)	(0.001)
Child’s age squared				−0.004**	−0.008**	−0.004**	−0.003**	−0.007**	−0.004**
				(0.000)	(0.0006)	(0.000)	(0.000)	(0.001)	(0.000)
Gender (male)				0.020**	0.020*	0.006**	0.023**	0.046**	0.008**
				(0.002)	(0.009)	(0.0009)	(0.002)	(0.010)	(0.001)
Birth order				0.005**	0.165**	0.012**	0.001	0.130**	0.014**
				(0.0008)	(0.005)	(0.0005)	(0.001)	(0.006)	(0.001)
Parents and Household-Level Controls									
Religion (Hindu = 1)							0.031**	0.199**	0.011**
							(0.004)	(0.017)	(0.002)
Low caste							−0.016**	−0.094**	−0.004**
							(0.002)	(0.013)	(0.001)
Middle caste							0.001	−0.027*	0.000
							(0.002)	(0.012)	(0.001)
Rural							0.014**	0.127**	0.009**
							(0.003)	(0.015)	(0.001)
Low wealth							−0.045**	−0.526**	−0.036**
							(0.003)	(0.019)	(0.002)
Medium wealth							−0.003	−0.190**	−0.019**
							(0.002)	(0.016)	(0.001)
Mother is illiterate							−0.025**	−0.229**	−0.018**
							(0.002)	(0.012)	(0.001)
Mother is primary schooled							0.012**	0.012	−0.003*
							(0.001)	(0.012)	(0.001)
Father is illiterate							−0.086**	−0.501**	−0.028**
							(0.003)	(0.013)	(0.001)
Father is primary schooled							−0.011**	−0.184**	−0.018**
							(0.002)	(0.010)	(0.001)
Mother’s age							0.017**	−0.095**	−0.010**
							(0.004)	(0.019)	(0.002)
Mother’s age squared							−0.000**	0.002**	0.000**
							(0.000)	(0.000)	(0.000)
Father’s age							0.003^†^	−0.018^†^	0.001
							(0.002)	(0.011)	(0.001)
Father’s age squared							−0.000*	0.000*	−0.000
							(0.000)	(0.000)	(0.000)
District Fixed Effects	Yes	Yes	Yes	Yes	Yes	Yes	Yes	Yes	Yes
*R* ^2^	.07	.007	.03	.11	.69	.14	.14	.71	.15
Number of Observations	393,510	393,510	345,985	393,510	393,510	345,985	393,510	393,510	345,985

*Notes:* Robust standard errors, clustered by district, are shown in parentheses. SES is measured by wealth index of the household. Family size is total number of 0- to 20-year-old children in the family at the time of the survey. Low caste is scheduled caste (SC) and scheduled tribe (ST) households, and the middle caste is other backward caste (OBC).

^†^ *p* ≤ .10; *p ≤ .05; **p ≤ .01

Recognizing the limitation of interpreting the OLS estimates in Table 3 as causal, we then proceed to estimate the same relationship using 2SLS. We estimate the models with and without controlling for SES. Because SES and child quality may be affected jointly by the quantity of children, controlling for SES would mean overadjustment in the model (Angrist and Pischke 2009).

We first check for the relevance condition in Table 4. From the first-stage regression, it follows that the instrument is highly significant and has a positive correlation with family size. The first row in Table 4 shows that family size increases by 0.22 children when the firstborn is a girl, and the effect is significant at the 1 % level of significance.

**Table 4:** 2SLS estimates of the effect of family size on education

	Instrument: Firstborn Girl					
	Ever Attended School		Years of Schooling		Currently Enrolled	
	(1)	(2)	(3)	(4)	(5)	(6)
First Stage	0.218**	0.219**	0.218**	0.219**	0.226**	0.228**
	(0.007)	(0.007)	(0.007)	(0.007)	(0.007)	(0.007)
Weak-Identification Tests						
Keibergen-Paap Wald *rk F* stat.	1,062.66	1,071.10	1,062.66	1,071.10	1,074.11	1,071.10
Cragg-Donald Wald *F* stat.	4,164.83	4,280.87	8,732.70	4,280.87	4,170.26	4,280.87
Weak-Instrument-Robust, Inference						
Anderson-Rubin *F* stat.	8.27	9.43	4.43	5.85	9.49	10.74
*p* value	.004	.002	.04	.016	.002	.001
Stock-Wright *S* stat.	8.94	9.16	5.46	5.71	10.48	10.49
*p* value	.003	.003	.02	.017	.001	.001
2SLS Results						
Family size	−0.017**	−0.017**	−0.071*	−0.079*	−0.011**	−0.011**
	(0.006)	(0.006)	(0.033)	(0.033)	(0.004)	(0.004)
Child-level controls						
Child’s age	0.080**	0.080**	0.808**	0.811**	0.060**	0.060**
	(0.003)	(0.003)	(0.015)	(0.015)	(0.001)	(0.001)
Child’s age squared	−0.003** (0.000)	−0.003** (0.000)	−0.007** (0.001)	−0.007** (0.001)	−0.004** (0.000)	−0.004** (0.000)
Gender (male)	0.023**	0.023**	0.086**	0.084**	0.009**	0.009**
	(0.002)	(0.002)	(0.011)	(0.011)	(0.001)	(0.001)
Birth order	0.001	0.001	0.043^†^	0.049*	0.012**	0.012**
	(0.004)	(0.004)	(0.023)	(0.022)	(0.002)	(0.002)
Parents and household-level controls						
Religion (Hindu = 1)	0.030** (0.005)	0.032** (0.005)	0.244** (0.022)	0.252** (0.022)	0.011** (0.002)	0.012** (0.002)
Low caste	−0.021**	−0.016**	−0.179**	−0.114**	−0.009**	−0.005**
	(0.003)	(0.003)	(0.015)	(0.014)	(0.001)	(0.001)
Middle caste	−0.001	0.001	−0.065**	−0.033*	−0.002	0.000
	(0.002)	(0.002)	(0.013)	(0.013)	(0.001)	(0.001)
Rural	0.003	0.014**	−0.016	0.133**	−0.001	0.009**
	(0.003)	(0.003)	(0.015)	(0.015)	(0.001)	(0.001)
Low wealth		−0.046**		−0.581**		−0.037**
		(0.004)		(0.023)		(0.002)
Medium wealth		−0.003		−0.221**		−0.020**
		(0.003)		(0.018)		(0.002)
Mother is illiterate	−0.035**	−0.025**	−0.378**	−0.259**	−0.026**	−0.019**
	(0.003)	(0.002)	(0.017)	(0.014)	(0.002)	(0.001)
Mother is primary schooled	0.007** (0.002)	0.012** (0.002)	−0.073** (0.014)	−0.002 (0.013)	−0.008** (0.001)	−0.003** (0.001)
Father is illiterate	−0.095**	−0.086**	−0.603**	−0.517**	−0.033**	−0.028**
	(0.003)	(0.003)	(0.015)	(0.014)	(0.001)	(0.001)
Father is primary schooled	−0.016** (0.002)	−0.011** (0.002)	−0.250** (0.011)	−0.194** (0.010)	−0.021** (0.001)	−0.018** (0.001)
Mother’s age	0.017**	0.017**	−0.101**	−0.100**	−0.010**	−0.010**
	(0.004)	(0.004)	(0.020)	(0.019)	(0.002)	(0.002)
Mother’s age squared	−0.000**	−0.000**	0.002**	0.002**	0.000**	0.000**
	(0.000)	(0.000)	(0.000)	(0.000)	(0.000)	(0.000)
Father’s age	0.003^†^	0.003^†^	−0.024*	−0.028*	0.001	0.000
	(0.002)	(0.002)	(0.011)	(0.011)	(0.001)	(0.001)
Father’s age squared	−0.000*	−0.000*	0.000*	0.000**	−0.000	−0.000
	(0.000)	(0.000)	(0.000)	(0.000)	(0.000)	(0.000)
SES	No	Yes	No	Yes	No	Yes
District Fixed Effect	Yes	Yes	Yes	Yes	Yes	Yes
*R* ^2^	.08	.09	.68	.68	.14	.14
Number of Observations	393,510	393,510	393,510	393,510	345985	345,985

*Notes:* Robust standard errors, clustered by district, are shown in parentheses. Family size is total number of 0- to 20-year-old children in the family at the time of the survey. SES is measured using the wealth index of the household. Low caste is scheduled caste (SC) and scheduled tribe (ST) households, and the middle caste is other backward caste (OBC).

^†^ *p* ≤ .10; *p ≤ .05; **p ≤ .01

The 2SLS results presented in Table 4 show a negative and significant impact of family size on children’s quality. The results show that inclusion of SES in the model does not change the main findings in a significant way. Results are qualitatively and quantitatively similar across models with and without SES. Therefore, our preferred estimates are from the model that controls for household SES. The estimates for ever being in school and current enrollment are negative and statistically significant, confirming that the detrimental effects of family size on children’s education comes from both not ever attending school and from dropping out of school along the way. Columns 2 and 6 show that the probability of ever attending school and being currently enrolled drop by 1.7 and 1.1 percentage points, respectively, when an additional sibling is added to the family. The magnitude of the effects is not very large, which may not be surprising given that Table 1 shows school attendance and current enrollment rate are approximately 90 % and 95 %, respectively, in our sample. These coefficients imply that having an extra sibling increases the probability of never attending school and not being enrolled in school at the time of survey by 1.9 % and 1.2 %, respectively. Next, we look at whether years of schooling are affected by larger family size (column 4). The 2SLS results for years of schooling indicate that an increase in household size of one extra child decreases the years of schooling by 0.08 compared with 0.2 when relying on OLS estimates, or by 2.6 % instead of 6.5 % at the mean years of schooling of 3.08 years. The impact on years of schooling is small but economically meaningful and comparable with other educational interventions in developing countries. At the mean family size of 3.54 in our sample, this translates to a reduction of 0.28 years of schooling in the average family, which is comparable with findings in other studies of education-specific policy interventions (Azam and Saing 2016; Duflo 2001).^9^ Our finding implies that population stabilization policy may be as effective as education policy in improving human capital in developing countries.

The IV results suggest that after we account for the endogeneity in family size, the 2SLS coefficients are smaller (or less negative) compared with the OLS estimates, implying that OLS coefficients overestimate the true trade-off and are biased toward finding effects that are too large. Thus, unobservable characteristics that drive parents to have big families also drive them to invest too little in their children.

Table 4 also reports the Kleibergen-Paap *rk* Wald test to detect whether the instrument suffers from a weak-intrument problem. Both the first-stage *F* statistic and Kleibergen-Paap *rk* Wald Statistic are significant, indicating that our analysis does not suffer from a weak-instrument problem. We also report the Anderson-Rubin *F* test Statistic and the Stock-Wright *S* statistic in Table 4 to confirm that our second-stage results are robust to weak-instrument inference.

### Potential Threats to Identification

Next, we focus on the second key assumption that having a first child who is a girl is unlikely to be correlated with other factors associated with educational outcomes. As noted earlier, one potential concern is the influence of sex-selective abortions. However, we have presented evidence from both our data and previous studies showing that first births are not subject to self-selective abortions. Moreover, although we find strong evidence of a Q-Q trade-off in Table 4 following the legal ban of abortions, panel A of Table 8 in the appendix shows no evidence of a trade-off in the period before the abortion ban.

Another potential concern is that the gender of the first child may be related to sibling sex composition in the household. Gender of the first child may affect not only family size but also the sex composition of the siblings because of son-preferring, differential stopping behaviour (SP-DSB) (Barcellos et al. 2014). In this case, π_1_ in Eq. (3) will capture the family size effect as well as the sibling sex composition effect. The empirical evidence on the effect of sibling sex composition on children’s education is ambiguous.^10^ In the context of developing countries, sibling rivalry or competition for limited resources may mean that having more male siblings reduces resources for girls, but the evidence is mixed.^11^ Rosenblum (2013b) showed that in India, girls in firstborn girl households are worse off than those in the firstborn boy households. By contrast, Makino (2012) found that boys in India are worse off when they have more brothers and are better off with more sisters, but also that the gender composition of siblings has no effect on girls’ outcomes.

We check for the existence of SP-DSB in our data and find that Indian households do engage in the son-biased stopping rule, as evident in the first two columns of Table 9 in the appendix. The first two columns show the results of regressing the total number of children in the family on the gender of the first child and different combinations of the first and second child’s gender. We find that a firstborn girl and firstborn and second-born girls (Girl, Girl) predict larger family sizes. Column 3 also shows that a firstborn girl increases the likelihood of more girls in the family. To address the concern of SP-DSB and the sibling sex composition effect, we include the number of girls as an additional control in our model. Because gender composition of siblings is also endogeneous, we instrument it by the interaction of the gender of the first child and mother’s age. Although we use this to instrument for gender composition in the household, Lee (2008) instead used the interaction of the gender of the first child with mother’s age as well as with mother’s education to instrument for the family size in a nonlinear model.^12^

Table 5 reports the 2SLS estimates after controlling for the number of girls in the household. The results of the augmented specification with additional control for number of girls are similar to the results in Table 4 but are generally larger. The impact of having one more sibling is to reduce years of schooling by 0.89 years. The results in Table 5 are somewhat noisy and are larger than even the OLS estimates. Moreover, because we recognize the difficulty in finding a good instrument for the sibling sex composition, we consider the model in Table 4 with the SES control as our preferred specification. Thus, in the rest of the analyses, we continue to estimate the specification in Table 4 with the SES control. However, given the mixed evidence on the impacts of sibling sex composition, we take the results without controls for sibling sex composition as potentially upper bounds of the effect of family size because family size in this specification could also be capturing the sibling sex composition effect on educational attainment.

**Table 5:** 2SLS estimates with control for sibling sex composition

	Instrument: Firstborn Girl		
	Ever Attended School	Years of Schooling	Currently Enrolled
	(1)	(2)	(3)
Family Size	−0.119*	−0.895**	−0.174**
	(0.049)	(0.268)	(0.047)
Child-Level Controls			
Child’s age	0.096**	0.939**	0.084**
	(0.008)	(0.042)	(0.007)
Child’s age squared	−0.004**	−0.009**	−0.004**
	(0.000)	(0.001)	(0.000)
Gender (male)	0.023**	0.082**	0.011**
	(0.002)	(0.012)	(0.002)
Birth order	0.054*	0.477**	0.094**
	(0.026)	(0.143)	(0.024)
Parents and Household-Level Controls			
Number of girls	0.030*	0.238**	0.049**
	(0.015)	(0.076)	(0.014)
Religion (Hindu = 1)	−0.009	−0.073	−0.052**
	(0.020)	(0.109)	(0.019)
Low caste	−0.000	0.007	0.020**
	(0.008)	(0.042)	(0.008)
Middle caste	0.006^†^	0.007	0.009**
	(0.003)	(0.018)	(0.003)
Rural	0.010**	0.095**	0.003
	(0.004)	(0.020)	(0.003)
Low wealth	−0.004	−0.250*	0.025
	(0.020)	(0.110)	(0.018)
Medium wealth	0.019*	−0.041	0.014
	(0.011)	(0.061)	(0.010)
Mother is illiterate	−0.002	−0.077	0.018^†^
	(0.011)	(0.061)	(0.011)
Mother is primary schooled	0.022**	0.081**	0.015**
	(0.005)	(0.031)	(0.005)
Father is illiterate	−0.074**	−0.417**	−0.012*
	(0.007)	(0.035)	(0.005)
Father is primary schooled	−0.004	−0.134**	−0.007^†^
	(0.004)	(0.022)	(0.004)
Mother’s age	0.017**	−0.101**	−0.012**
	(0.004)	(0.019)	(0.003)
Mother’s age squared	−0.000**	0.001**	0.000
	(0.000)	(0.000)	(0.000)
Father’s age	0.010**	0.027	0.011**
	(0.004)	(0.021)	(0.003)
Father’s age squared	−0.000**	−0.000	−0.000**
	(0.000)	(0.000)	(0.000)
District Fixed Effects	Yes	Yes	Yes
Number of Observations	393,510	393,510	345,985

*Notes:* Robust standard errors, clustered by district, are shown in parentheses. Family size is total number of 0- to 20-year-old children in the family at the time of the survey. Sex composition of the household (number of girls) is instrumented by the interaction of gender of the first child and mother’s age. SES is measured using the wealth index of the household. Low caste is scheduled caste (SC) and scheduled tribe (ST) households, and the middle caste is other backward caste (OBC).

^†^ *p* ≤ .10; *p ≤ .05; **p ≤ .01

The gender of the firstborn could also be related to omitted factors affecting education if the likelihood of having a firstborn girl increases the probability of mothers’ employment and propensity to accumulate assets to pay the dowries for daughters’ marriage. We check this possibility by estimating a regression of the likelihood that a mother was employed in the last 7 days or 12 months on an indicator that the firstborn is a girl. We find no significant effect, confirming that mother’s higher probability of employment is not contaminating our main results (see columns 1 and 2 in Table 10 in the appendix). Households with a firstborn girl may also save more money to pay for a daughter’s dowry, which may reduce education regardless of family size. Our data set does not have detailed information on saving behavior of the households. However, we take advantage of information collected on landownership and other physical assets that may proxy for household’s savings and estimate the effect of firstborn girl on ownership of land and other physical assets. We find no differential effect on ownership of these assets by gender of the first child, which again confirms that our main results are not driven by these other omitted factors (see columns 3 and 4 in Table 10 in the appendix).

Rosenblum (2013b) also noted that SP-DSB may lower survival rates for girls in India.^13^ Because we observe only surviving girls who are firstborn, this may generate positive selection in our observed sample, implying that we may be observing only very strong girls with better health and better educational outcomes. However, if one believes that other younger girl siblings following the firstborn girl are also likely to be strong, then this would bias the estimates downward because the strong children in these households would grow up with more siblings but also would likely do better in school. This may violate the exclusion restriction of the instrument. Another reason why the exclusion restriction may be problematic is the excess mortality among adult women due to son preference. A study by Milazzo (2014) found that having a firstborn who is a girl increases maternal and adult mortality after age 30. Because of son preference, these women are more likely to engage in fertility behavior that negatively affects their health and are thus less likely to survive. If the death of the mother affects the educational outcomes of children in these households, this would amplify the educational impact attributed to family size. Because we focus on mothers under age 35, our analysis is unlikely to suffer from this type of bias. However, we conduct a robustness check by further limiting the analysis to mothers under age 30, given that younger mothers’ mortality is not affected by having a firstborn girl as per the findings in Milazzo (2014), and our results in panel B of Table 8 in the appendix are substantively identical to the main findings in Table 4.

### Alternative Definitions of Family Size

In our main models, we restrict the family size variable to school-aged children who are 0–20 years of age. In sensitivity analyses, we further restrict our analysis to households in which the oldest child is younger than 18 years and 15 years to check the robustness of our results for different ages of school-going children. Additionally, we also relax this sample restriction altogether and consider all *resident* children irrespective of age as measure of family size. We present results from these alternative definitions of family size in panels C, D, and E in Table 8 in the appendix. The results show that parents continue to make similar trade-offs when the oldest child in the household is restricted to those aged 15 and 18 years or younger, or when all resident children in the family are included.

## Heterogeneous Results

### Caste Differences in the Q-Q Trade-off

Given the disadvantaged situation of lower castes in India, one may expect lower castes to have less access to schools than higher castes.

We capture the heterogeneity in the Q-Q trade-off across different caste categories by estimating the specification with the SES control in Table 4 separately for different caste categories. Results in columns 1–3 of Table 6 show that after family size is instrumented,^14^ the effect of family size on the likelihood of ever attending school and actual years of schooling is greatest for low-caste individuals. For example, having an extra sibling in low-caste households reduces the years of schooling by 0.16 of a year for a single child and by close to one-half of a year for an average family with three children. This compares with the average effect of one-quarter of a year for a child in a middle-caste household. It also compares with no effect on children of high-caste households. Because the average years of schooling among low-caste children is 2.8 years, this translates to a 5.7 % reduction in years of schooling due to having an additional child in the family. Similarly, having an extra sibling in low-caste households reduces the likelihood of ever attending school by 3.6 percentage points, which is double what we found in Table 4 for the full sample. This also compares with no effect on middle- and high-caste households. We observe no effects on high-caste households for current enrollment. By contrast, growing up with an extra sibling reduces the likelihood of being currently enrolled by between 0.006 and 0.019 for children in low- and middle-caste households, although the effects on low-caste households are not statistically significant in this case. These results suggest that family size has a more negative impact on lower-caste families that cannot overcome educational and liquidity constraints.^15^

**Table 6:** 2SLS estimates by caste and residence

	Low Caste	Middle Caste	High Caste	Rural	Urban
Dependent Variables	(1)	(2)	(3)	(4)	(5)
Ever Attended School	−0.036**	−0.012	0.006	−0.018**	−0.021
	(0.010)	(0.009)	(0.011)	(0.007)	(0.014)
Years of Schooling	−0.162**	−0.085^†^	0.094	−0.105**	−0.047
	(0.054)	(0.050)	(0.064)	(0.035)	(0.087)
Currently Enrolled	−0.006	−0.019**	−0.008	−0.010**	−0.026**
	(0.006)	(0.005)	(0.007)	(0.004)	(0.010)
Children’s Controls	Yes	Yes	Yes	Yes	Yes
Parent’ Controls	Yes	Yes	Yes	Yes	Yes
District Fixed Effect	Yes	Yes	Yes	Yes	Yes

*Notes:* Robust standard errors, clustered by district, are shown in parentheses. Children’s controls include age, age squared, gender, and birth order. Parents’ controls include age, age squared, religion, caste, rural dummy variables, education levels of father and mother, and household’s SES. Family size is total number of 0- to 20-year-old children in the family at the time of the survey.

^†^ *p* ≤ .10; **p ≤ .01

### Rural-Urban Differences in the Q-Q Trade-off

Given the lack of good public schools in rural areas in India, we may expect for the Q-Q trade-off to be greater in rural than urban areas. Indeed, there are large rural-urban gaps in educational attainment. For our sample children, the primary school completion rate is 35 % in rural areas and 41 % in urban areas.

Columns 4 and 5 in Table 6 show the 2SLS results for rural and urban areas, respectively. Indeed, the impact of having larger families is larger and statistically significant in rural compared with urban areas, suggesting that the Q-Q trade-off is more pronounced in rural India. The coefficients in column 4 suggest that having an extra child reduces the likelihood of ever attending school by 1.8 percentage points and years of schooling by one-tenth of a year in rural households compared with urban households. At the mean years of schooling in rural areas of 2.9 years, the Q-Q coefficient implies a reduction of 3.7 %. These findings are similar to those in Li et al. (2008), who reported stronger Q-Q trade-offs in rural areas in China. Surprisingly, the coefficient for current enrollment is higher in urban areas compared with rural areas.

### Wealth and the Q-Q Trade-off

The severity of the trade-off may also differ by household wealth. Wealthier households are less likely to be subject to credit constraints when making the choice between the number of children and the educational opportunities offered to each child. We classify households as poor and nonpoor based on their wealth level. Households in low- and middle-wealth categories are grouped as poor, and households in high-wealth groups are grouped as nonpoor. The results in columns 1 and 2 of Table 7 show the 2SLS results by household wealth levels.^16^ The effect of having an extra child on the likelihood of going to school, years of schooling, and on the likelihood of being currently enrolled in school are all greatest for children in poor households than for those in nonpoor households. Having an extra sibling reduces the likelihood of attending school and being currently enrolled by 3.9 percentage points and 1.8 percentage points, respectively. By contrast, children in the nonpoor households experience no Q-Q trade-off in ever attending school, and the effect on current enrollment is less than one-half of that found for children in poor households. For years of schooling, having an extra sibling reduces years of schooling by slightly more than one-quarter of a year for poor households but has no effect on nonpoor households. The average years of schooling among poor children is only 2.5 years, so the Q-Q coefficient for years of schooling implies a big impact in percentage terms: having an additional child reduces years of schooling for a poor child by 11 %.

**Table 7:** 2SLS estimates by household wealth and mother’s education

	Household Wealth		Mother’s Education	
	Poor	Nonpoor	Primary and Less	More Than Primary
Dependent Variables	(1)	(2)	(3)	(4)
Ever Attended School	−0.039**	−0.005	−0.029**	0.006
	(0.011)	(0.005)	(0.007)	(0.006)
Years of Schooling	−0.262**	0.036	−0.190**	0.108*
	(0.052)	(0.035)	(0.039)	(0.050)
Currently Enrolled	−0.018**	−0.007^†^	−0.018**	−0.005
	(0.006)	(0.004)	(0.004)	(0.004)
Children’s Controls	Yes	Yes	Yes	Yes
Parent’ Controls	Yes	Yes	Yes	Yes
District Fixed Effect	Yes	Yes	Yes	Yes

*Notes:* Robust standard errors, clustered by district, are shown in parentheses. Children’s controls include age, age squared, gender, and birth order. Parents’ controls include age, age squared, religion, caste, rural dummy variables, education levels of father and mother, and household’s SES. Family size is total number of 0- to 20-year-old children in the family at the time of the survey. Poor is defined as households in bottom two wealth quantiles. Primary schooling is defined as having completed five years of schooling.

^†^ *p* ≤ .10; *p ≤ .05; **p ≤ .01

## Does Mother’s Educational Attainment Affect the Q-Q Trade-off?

Mothers play a key household role by making expenditure decisions and by providing a supportive environment for children. Also, less-educated mothers will generally be less able to provide support for children in their studies, possibly leading to bigger Q-Q trade-offs.

Columns 3 and 4 in Table 7 show the coefficients of the 2SLS model for mothers with primary and less than primary schooling and for mothers with more than primary schooling. The results show that the detrimental effects of having an extra child on educational attainment are greatest for children of low-educated mothers. The effect of having an extra sibling on years of schooling for the children of low-educated mothers is one-fifth of a year and statistically significant. By contrast, the impact of family size on years of schooling for children of mothers with more than primary schooling is one-tenth of a year. Similarly, having an extra sibling reduces the likelihood of ever having been enrolled and being currently enrolled by 2.9 and 1.8 percentage points, respectively, in households of low-educated mothers. By contrast, there are no significant impacts on attendance for children of more-educated mothers.

All in all, the Q-Q trade-offs are more pronounced among lower-caste, rural, and poorer households, as well as among households with less-educated mothers, probably because these households face the greatest credit constraints, attend worse public school systems, and are less able to compensate for bad schooling by educating their children at home or by relying on private tutoring.

## Conclusions

In this study, we use nationally representative household data to test the empirical validity of the Q-Q trade-off in India. A strong preference for sons over daughters in Indian society allows us to use gender of the first child as an instrument to test the Q-Q trade-off. We find that family size has significant negative impacts on educational outcomes of children. Although our results may be an upper estimate of the impact of family size, we find that having an additional sibling can reduce average years of schooling by close to one-quarter of a year and reduce attendance by 1 to 2 percentage points. These results are modest but compare in magnitude with those of school construction and the provision of additional resources to schools (Azam and Saing 2016; Duflo 2001).

Importantly, we find evidence of more pronounced Q-Q trade-offs among rural, low-caste, and poorer households, and for less-educated mothers, all of which are likely to face greater budget constraints and be exposed to lower-quality public schools. Because the majority of large families in developing countries are poor, less educated, and resource constrained, our findings can help us better understand why poverty persists. Improving access and uptake of family planning methods and public policies aimed at increasing awareness about the benefits of having a smaller family may help weaken the severity of the trade-off while helping poor families increase educational attainment and, in turn, move them out of poverty. Furthermore, policy-makers in developing countries can supplement family planning policies with more investment in education in regions and households for which the trade-off is severe in order to mitigate the adverse impacts of larger families. Finally, policies should be designed to weaken son bias. For example, extending the inheritance rights to daughters and establishing a welfare system or an old-age social security program would reduce the need to rely on children for social security in old age.

### Electronic supplementary material

ESM 1 (DOCX 40 kb)

## Appendix

**Table 8:** Robustness checks with pre-abortion ban period, for younger mothers, and alternative definitions of family size

	Instrument: First Born Girl		
	Ever Attended School	Years of Schooling	Currently Enrolled
	(1)	(2)	(3)
A. Analysis on Pre-Ban Data (NFHS, 1992–1993)			
Family size	––	−0.166	−0.022
	––	(0.730)	(0.020)
B. For Mothers Below 30 Years of Age			
Family size	−0.026**	−0.126*	−0.008*
	(0.011)	(0.038)	(0.004)
C. Oldest Child Is 18 Years Old			
Family size	−0.016**	−0.095**	−0.016**
	(0.006)	(0.031)	(0.004)
D. Oldest Child Is 15 Years Old			
Family size	−0.013*	−0.094**	−0.018**
	(0.007)	(0.031)	(0.004)
E. Number of Children Home at Survey			
Family size	−0.017*	−0.079**	−0.011**
	(0.006)	(0.033)	(0.004)
Children’s Controls	Yes	Yes	Yes
Parents’ Controls	Yes	Yes	Yes

*Notes:* Robust standard errors, clustered by district, are shown in parentheses. Children’s controls include age, age squared, gender, and birth order. Parents’ controls include age, age squared, religion, caste, rural dummy variables, education levels of father and mother, and household’s SES. Family size in panel E is defined as total numer of children at home at the time of the survey irrespective of their age. Panel A includes state fixed effects, and panels B–E include district fixed effects.

**p* ≤ .05; ***p* ≤ .01

**Table 9:** Son-preferring differential stopping behavior (SP-DSB)

	Total Number of Children	Total Number of Children	Total Number of Female Children
	(1)	(2)	(3)
Firstborn Girl	0.219**		0.752**
	(0.007)		(0.005)
(Girl, Girl)		0.452**	
		(0.010)	
(Boy, Boy)		−0.089**	
		(0.007)	
Children’s Controls	Yes	Yes	Yes
Parents’ Controls	Yes	Yes	Yes
District Fixed Effect	Yes	Yes	Yes

*Notes:* Robust standard errors, clustered by district, are shown in parentheses. Children’s controls include age, age squared, gender, and birth order. Parents’ controls include age, age squared, religion, caste, rural dummy variables, education levels of father and mother, and household’s SES.

***p* ≤ .01

**Table 10:** Effect of gender of first child on mother’s employment and asset ownership

	Worked in Last 7 Days	Worked in Last 12 Months	Land Ownership	Asset Index
	(1)	(2)	(3)	(4)
Firstborn Girl	0.001	−0.003^†^	−0.003	−0.0004
	(0.002)	(0.001)	(0.002)	(0.003)
SES	No	No	No	No
Parents’ Controls	Yes	Yes	Yes	Yes
District Fixed Effect	Yes	Yes	Yes	Yes
*R* ^2^	.21	.07	.23	.63
Number of Observations	152,477	152,477	162,619	162,592

*Notes:* Robust standard errors, clustered by district, are shown in parentheses. Columns 1 and 2 are at mother’s level; columns 3 and 4 are at the household level. The regression includes the following control variables: household size, mother’s and husband’s age and age squared, religion, caste, rural dummy variables, and education levels of mother and the husband. SES is measured using wealth index of the household.

^†^ *p* ≤ .10

## Footnotes

1 Lee (2008) also used four additional IVs, including interactions of “first girl” with exogenous variables (such as mother’s age and presence of grandparents), to estimate the nonlinear effects of child quantity on quality.

2 Contrary to other studies, Lee (2008) measured impact of family size on educational expenditures in South Korea. South Korea is a country where educational expenditures are substantial, but time investments may be more important in a country like India where education is mostly publicly funded. Moreover, in the end, it is the educational attainment (and not the amount of financial resources spent) that matters in terms of overall family well-being and human capital investments in the country.

3 We restrict the family size to children aged 0–20 to focus our analysis on school-age children. Older children (21 years of age and older) are more likely to have already completed high school and to be active in the labor market, so their inclusion in the sample may dilute the true effect of family size on the educational attainment of school-going children. Furthermore, there are few households that have children over 21, and we do not lose many observations by restricting the family size to those including children younger than 20 years old. Nonetheless, we later present results with robustness tests including children of all ages residing in the home and all children under 15 and 18 years of age to focus on a narrower group of school-age children.

4 Since we observe only surviving children in the data, selection due to observing only surviving children is likely to generate a downward bias in our estimates of the Q-Q trade-off estimate because surviving children are observed in smaller families and are also likely to be healthier and better performing children in school.

5 In the absence of any interventions, the probability of having a son is approximately .512, and this probability is independent of genetic factors (Ben-Porath and Welch 1976; Jacobsen et al. 1999).

6 We drop individuals who are sons- or daughters-in-law, grandchildren, parents, parents-in-law, brothers, sisters, brothers- or sisters-in-law, nieces or nephews, and other relatives.

7 Urban areas are defined as having a minimum population size of 50,000 or as having at least 75 % of the male working population engaged in nonagricultural activities, or as having a population density of at least 1,000 per square mile. All residual areas that do not meet these criteria are classified as rural areas. The majority of the rural population are engaged in agricultural activities.

8 By combining household amenities, assets, and durables, the DLHS data were used to compute a wealth index divided into quartiles. The principle of factor loading to amenities, assets, and durables derived by factor analysis is used for the computation of the wealth index. Households are categorized from the poorest to the richest groups corresponding to the lowest to the highest quartiles.

9 In an evaluation of the District Primary Education Program (DPEP) in India, Azam and Saing (2016) found that increasing resources in school led to an increase of between 0.1 and 0.2 years of schooling. Similarly, Duflo (2001) found that each primary school constructed per 1,000 children under the Sekolah Dasar INPRES program in Indonesia during 1973–1979 led to an average increase of 0.12 to 0.19 years of education in Indonesia.

10 In the United States, Butcher and Case (1994) found that the sibling sex composition—and, in particular, having more male siblings—increases the educational attainment of the girls but not of the boys. However, Kaestner (1997) suggested that sibling sex composition is not a significant factor in explaining the difference in educational attainment in the United States.

11 Akresh and Edmonds (2011) found evidence of sibling rivalry in Burkina Faso when households face constraints.

12 Lee (2008) argued that because the age profile of fertility differs by the gender of the first child in South Korea, interaction of mother’s age with the gender of the first child can be used as an additional instrument for family size. Furthermore, Lee’s study includes interaction of mother’s education with the gender of the first child because less-educated mothers are more likely to have stronger preference for sons in Korea.

13 Similarly, Hu and Schlosser (2015) found that sex-selective abortion reduces malnutrition for surviving girls.

14 The first stage is similar for different households from different castes, with different wealth levels, with mothers of varying educational levels, and for urban and rural households.

15 The impacts of all the other control variables on educational attainment in the specifications in Table 6 are reported in Online Resource 1, Table S1.

16 The full results are included in Online Resource 1, Table S2.
