# Supplementary material for: Preference for Boys, Family Size, and Educational Attainment in India
Source: Demography. 2017 May 8;54(3):835–59. doi: 10.1007/s13524-017-0575-1 (PMC5486858; doi:10.1007/s13524-017-0575-1)
Supplement: Supplementary file 1 — (DOCX 40 kb) [file 13524_2017_575_MOESM1_ESM.docx]

**Online Resource 1**

**Preference for Boys, Family Size, and Educational Attainment in India**

Adriana D. Kugler and Santosh Kumar

| **Table S1** 2SLS estimates by caste and residence | | | | | | | | | | | | | | | |
| --- | --- | --- | --- | --- | --- | --- | --- | --- | --- | --- | --- | --- | --- | --- | --- |
|  | By caste | | | | | | | | | By residence | | | | | |
|  | Low caste | | | Middle caste | | | High caste | | | Rural | | | Urban | | |
|  | Ever attended school | Years of schooling | Currently enrolled | Ever attended school | Years of schooling | Currently enrolled | Ever attended school | Years of schooling | Currently enrolled | Ever attended school | Years of schooling | Currently enrolled | Ever attended school | Years of schooling | Currently enrolled |
|  |  |  |  |  |  |  |  |  |  |  |  |  |  |  |  |
| Family Size | -0.036*** | -0.162*** | -0.006 | -0.012 | -0.085* | -0.019*** | 0.006 | 0.094 | -0.008 | -0.018*** | -0.105*** | -0.010*** | -0.021 | -0.047 | -0.026*** |
|  | 0.010 | 0.054 | 0.006 | 0.009 | 0.050 | 0.005 | 0.011 | 0.064 | 0.007 | 0.007 | 0.035 | 0.004 | 0.014 | 0.087 | 0.010 |
| **Child-level controls** | | | |  |  |  |  |  |  |  |  |  |  |  |  |
| Child’s age | 0.094*** | 0.841*** | 0.060*** | 0.082*** | 0.789*** | 0.063*** | 0.053*** | 0.814*** | 0.055*** | 0.086*** | 0.803*** | 0.062*** | 0.055*** | 0.864*** | 0.055*** |
|  | 0.004 | 0.020 | 0.002 | 0.003 | 0.021 | 0.002 | 0.003 | 0.020 | 0.002 | 0.003 | 0.016 | 0.002 | 0.003 | 0.021 | 0.003 |
| Child’s age squared | -0.004*** | -0.010*** | -0.004*** | -0.003*** | -0.006*** | -0.004*** | -0.002*** | -0.004*** | -0.003*** | -0.004*** | -0.008*** | -0.004*** | -0.002*** | -0.005*** | -0.003*** |
|  | 0.000 | 0.001 | 0.000 | 0.000 | 0.001 | 0.000 | 0.000 | 0.001 | 0.000 | 0.000 | 0.001 | 0.000 | 0.000 | 0.001 | 0.000 |
| Gender (male) | 0.023*** | 0.093*** | 0.010*** | 0.029*** | 0.119*** | 0.009*** | 0.015*** | 0.014 | 0.005** | 0.027*** | 0.118*** | 0.012*** | 0.006* | -0.085*** | -0.006** |
|  | 0.003 | 0.015 | 0.002 | 0.004 | 0.017 | 0.002 | 0.004 | 0.019 | 0.002 | 0.003 | 0.012 | 0.001 | 0.004 | 0.021 | 0.003 |
| Birth order | 0.015** | 0.083** | 0.009** | -0.004 | 0.067** | 0.016*** | -0.015** | -0.065 | 0.009** | 0.001 | 0.053** | 0.011*** | 0.004 | 0.051 | 0.021*** |
|  | 0.007 | 0.036 | 0.004 | 0.006 | 0.033 | 0.003 | 0.007 | 0.042 | 0.004 | 0.004 | 0.024 | 0.003 | 0.009 | 0.055 | 0.006 |
| **Parents and household-level controls** | | | | |  |  |  |  |  |  |  |  |  |  |  |
| Religion (Hindu=1) | 0.012** | 0.045 | -0.001 | 0.054*** | 0.324*** | 0.015*** | 0.040*** | 0.370*** | 0.015*** | 0.035*** | 0.240*** | 0.011*** | 0.018** | 0.226*** | 0.009* |
|  | 0.006 | 0.028 | 0.003 | 0.008 | 0.035 | 0.004 | 0.008 | 0.043 | 0.004 | 0.006 | 0.025 | 0.002 | 0.007 | 0.041 | 0.005 |
| Rural | 0.009** | 0.107*** | 0.008*** | 0.020*** | 0.136*** | 0.007*** | 0.010*** | 0.117*** | 0.008*** |  |  |  |  |  |  |
|  | 0.004 | 0.024 | 0.002 | 0.004 | 0.020 | 0.002 | 0.004 | 0.025 | 0.002 |  |  |  |  |  |  |
| Low caste |  |  |  |  |  |  |  |  |  | -0.019*** | -0.131*** | -0.006*** | 0.000 | -0.049* | -0.001 |
|  |  |  |  |  |  |  |  |  |  | 0.003 | 0.017 | 0.002 | 0.004 | 0.026 | 0.003 |
| Middle caste |  |  |  |  |  |  |  |  |  | 0.000 | -0.042*** | -0.000 | 0.000 | -0.016 | 0.002 |
|  |  |  |  |  |  |  |  |  |  | 0.003 | 0.015 | 0.001 | 0.003 | 0.020 | 0.002 |
| Low wealth | -0.031*** | -0.643*** | -0.043*** | -0.058*** | -0.555*** | -0.034*** | -0.051*** | -0.550*** | -0.032*** | -0.026*** | -0.499*** | -0.035*** | -0.100*** | -0.657*** | -0.034*** |
|  | 0.006 | 0.038 | 0.004 | 0.005 | 0.033 | 0.003 | 0.007 | 0.044 | 0.004 | 0.004 | 0.026 | 0.002 | 0.010 | 0.053 | 0.006 |
| Medium wealth | 0.009** | -0.256*** | -0.021*** | -0.013*** | -0.209*** | -0.018*** | -0.016*** | -0.244*** | -0.020*** | 0.012*** | -0.143*** | -0.016*** | -0.02*** | -0.255*** | -0.017*** |
|  | 0.004 | 0.030 | 0.003 | 0.003 | 0.025 | 0.002 | 0.004 | 0.026 | 0.003 | 0.002 | 0.021 | 0.002 | 0.004 | 0.028 | 0.003 |
| Mother is illiterate | -0.024*** | -0.211*** | -0.018*** | -0.023*** | -0.236*** | -0.018*** | -0.029*** | -0.330*** | -0.015*** | -0.022*** | -0.217*** | -0.017*** | -0.029*** | -0.382*** | -0.020*** |
|  | 0.003 | 0.022 | 0.002 | 0.003 | 0.020 | 0.002 | 0.004 | 0.029 | 0.003 | 0.002 | 0.016 | 0.002 | 0.005 | 0.031 | 0.004 |
| Mother is primary schooled | 0.008*** | -0.009 | -0.003 | 0.014*** | 0.027 | -0.003* | 0.009*** | -0.051** | -0.003 | 0.012*** | 0.021 | -0.001 | 0.014*** | -0.074*** | -0.006** |
|  | 0.003 | 0.023 | 0.002 | 0.002 | 0.017 | 0.002 | 0.003 | 0.021 | 0.002 | 0.002 | 0.014 | 0.001 | 0.003 | 0.024 | 0.003 |
| Father is illiterate | -0.091*** | -0.483*** | -0.028*** | -0.081*** | -0.499*** | -0.025*** | -0.066*** | -0.525*** | -0.031*** | -0.087*** | -0.502*** | -0.026*** | -0.069*** | -0.507*** | -0.036*** |
|  | 0.004 | 0.020 | 0.002 | 0.004 | 0.018 | 0.002 | 0.005 | 0.029 | 0.003 | 0.003 | 0.014 | 0.001 | 0.006 | 0.032 | 0.004 |
| Father is primary schooled | -0.010*** | -0.153*** | -0.018*** | -0.012*** | -0.196*** | -0.014*** | -0.014*** | -0.243*** | -0.024*** | -0.011*** | -0.185*** | -0.017*** | -0.009** | -0.199*** | -0.016*** |
|  | 0.003 | 0.015 | 0.002 | 0.002 | 0.015 | 0.002 | 0.003 | 0.023 | 0.003 | 0.002 | 0.011 | 0.001 | 0.003 | 0.025 | 0.003 |
| Mother’s age | 0.015*** | -0.080*** | -0.013*** | 0.010* | -0.013 | -0.002 | 0.034*** | -0.244*** | -0.017*** | 0.016*** | -0.065*** | -0.009*** | 0.019** | -0.197*** | -0.014*** |
|  | 0.006 | 0.025 | 0.003 | 0.005 | 0.028 | 0.003 | 0.007 | 0.037 | 0.003 | 0.004 | 0.021 | 0.002 | 0.007 | 0.033 | 0.004 |
| Mother’s age | -0.000*** | 0.002*** | 0.000*** | -0.000 | 0.000 | 0.000 | -0.001*** | 0.004*** | 0.000*** | -0.000*** | 0.001*** | 0.000*** | -0.000** | 0.003*** | 0.000*** |
| squared | 0.000 | 0.000 | 0.000 | 0.000 | 0.000 | 0.000 | 0.000 | 0.001 | 0.000 | 0.000 | 0.000 | 0.000 | 0.000 | 0.001 | 0.000 |
| Father’s age | 0.003 | -0.024 | 0.000 | 0.004 | -0.038** | 0.000 | 0.002 | 0.050** | 0.004* | 0.003* | -0.009 | 0.001 | 0.001 | -0.066*** | -0.001 |
|  | 0.003 | 0.016 | 0.002 | 0.003 | 0.016 | 0.002 | 0.003 | 0.023 | 0.002 | 0.002 | 0.012 | 0.001 | 0.003 | 0.020 | 0.002 |
| Father’s age | -0.000 | 0.000 | -0.000 | -0.000* | 0.001** | -0.000 | -0.000 | -0.001** | -0.000* | -0.000** | 0.000 | -0.000* | -0.000 | 0.001*** | 0.000 |
| squared | 0.000 | 0.000 | 0.000 | 0.000 | 0.000 | 0.000 | 0.000 | 0.000 | 0.000 | 0.000 | 0.000 | 0.000 | 0.000 | 0.000 | 0.000 |
| Notes: Robust standard errors, clustered by district, are shown in parentheses. Family size is total number of 0-20 year old children in the family at the time of the survey. Low caste is scheduled caste and scheduled tribe households, and the middle caste is other backward caste. All models include district fixed effects.  ^†^*p* ≤ .10; **p* ≤ .05; ***p* ≤ .01 | | | | | | | | | | | | | | | |

| **Table S2** 2SLS estimates by household wealth and mother’s education | | | | | | | | | | | | |
| --- | --- | --- | --- | --- | --- | --- | --- | --- | --- | --- | --- | --- |
|  | By household’s wealth | | | | | | By mother’s education | | | | | |
|  | Non-Poor | | | Poor | | | Primary and less schooling | | | More than primary schooling | | |
|  | Ever attended school | Years of schooling | Currently enrolled | Ever attended school | Years of schooling | Currently enrolled | Ever attended school | Years of schooling | Currently enrolled | Ever attended school | Years of schooling | Currently enrolled |
| Family Size | -0.005 | 0.036 | -0.007* | -0.039*** | -0.262*** | -0.018*** | -0.029*** | -0.190*** | -0.018*** | 0.006 | 0.108** | -0.005 |
|  | 0.005 | 0.035 | 0.004 | 0.011 | 0.052 | 0.006 | 0.007 | 0.039 | 0.004 | 0.006 | 0.050 | 0.004 |
| **Child-level controls** | | | |  |  |  |  |  |  |  |  |  |
| Child’s age | 0.053*** | 0.892*** | 0.053*** | 0.114*** | 0.804*** | 0.074*** | 0.094*** | 0.783*** | 0.069*** | 0.043*** | 0.747*** | 0.027*** |
|  | 0.002 | 0.014 | 0.002 | 0.004 | 0.021 | 0.002 | 0.003 | 0.017 | 0.002 | 0.002 | 0.015 | 0.001 |
| Child’s age squared | -0.002*** | -0.007*** | -0.003*** | -0.005*** | -0.011*** | -0.005*** | -0.004*** | -0.008*** | -0.004*** | -0.002*** | 0.003*** | -0.002*** |
|  | 0.000 | 0.001 | 0.000 | 0.000 | 0.001 | 0.000 | 0.000 | 0.001 | 0.000 | 0.000 | 0.001 | 0.000 |
| Gender (male) | 0.009*** | 0.005 | 0.008*** | 0.037*** | 0.175*** | 0.012*** | 0.030*** | 0.128*** | 0.012*** | 0.002 | -0.052*** | 0.000 |
|  | 0.002 | 0.012 | 0.001 | 0.004 | 0.015 | 0.002 | 0.003 | 0.013 | 0.002 | 0.002 | 0.014 | 0.001 |
| Birth order | -0.004 | -0.047** | 0.007*** | 0.014* | 0.138*** | 0.016*** | 0.007 | 0.089*** | 0.015*** | -0.008** | -0.123*** | 0.003 |
|  | 0.003 | 0.022 | 0.003 | 0.008 | 0.037 | 0.004 | 0.005 | 0.026 | 0.003 | 0.004 | 0.031 | 0.002 |
| **Parents and household-level controls** | | | | |  |  |  |  |  |  |  |  |
| Religion (Hindu=1) | 0.023*** | 0.242*** | 0.014*** | 0.046*** | 0.228*** | 0.008** | 0.037*** | 0.251*** | 0.012*** | 0.010*** | 0.148*** | 0.007*** |
|  | 0.004 | 0.022 | 0.002 | 0.009 | 0.036 | 0.004 | 0.006 | 0.027 | 0.003 | 0.003 | 0.023 | 0.002 |
| Rural | 0.010*** | 0.113*** | 0.007*** | 0.047*** | 0.220*** | 0.016*** | 0.025*** | 0.209*** | 0.014*** | -0.000 | 0.015 | 0.003** |
|  | 0.002 | 0.015 | 0.001 | 0.009 | 0.034 | 0.004 | 0.004 | 0.020 | 0.002 | 0.002 | 0.013 | 0.001 |
| Low caste | -0.004** | -0.087*** | -0.002 | -0.029*** | -0.156*** | -0.009*** | -0.018*** | -0.124*** | -0.006*** | -0.007*** | -0.080*** | 0.000 |
|  | 0.002 | 0.016 | 0.002 | 0.005 | 0.023 | 0.003 | 0.003 | 0.017 | 0.002 | 0.002 | 0.017 | 0.001 |
| Middle caste | -0.004* | -0.047*** | 0.001 | -0.002 | -0.051** | -0.001 | 0.003 | -0.033** | -0.001 | -0.005*** | -0.030** | 0.001 |
|  | 0.002 | 0.013 | 0.001 | 0.004 | 0.022 | 0.002 | 0.003 | 0.016 | 0.002 | 0.001 | 0.013 | 0.001 |
| Low wealth |  |  |  |  |  |  | -0.063*** | -0.794*** | -0.054*** | -0.022*** | -0.320*** | -0.019*** |
|  |  |  |  |  |  |  | 0.005 | 0.032 | 0.003 | 0.003 | 0.028 | 0.002 |
| Medium wealth |  |  |  |  |  |  | -0.017*** | -0.398*** | -0.034*** | -0.008*** | -0.135*** | -0.010*** |
|  |  |  |  |  |  |  | 0.004 | 0.026 | 0.003 | 0.002 | 0.017 | 0.001 |
| Mother is illiterate | -0.024*** | -0.317*** | -0.020*** | -0.039*** | -0.283*** | -0.020*** |  |  |  |  |  |  |
|  | 0.002 | 0.017 | 0.002 | 0.004 | 0.022 | 0.002 |  |  |  |  |  |  |
| Mother is primary schooled | 0.003** | -0.072*** | -0.005*** | 0.005* | -0.043** | -0.005** |  |  |  |  |  |  |
|  | 0.001 | 0.014 | 0.001 | 0.003 | 0.020 | 0.002 |  |  |  |  |  |  |
| Father is illiterate | -0.046*** | -0.451*** | -0.032*** | -0.104*** | -0.524*** | -0.025*** | -0.090*** | -0.512*** | -0.028*** | -0.018*** | -0.269*** | -0.016*** |
|  | 0.003 | 0.018 | 0.002 | 0.004 | 0.017 | 0.002 | 0.003 | 0.014 | 0.001 | 0.003 | 0.029 | 0.003 |
| Father is primary schooled | -0.011*** | -0.213*** | -0.018*** | -0.017*** | -0.191*** | -0.018*** | -0.014*** | -0.199*** | -0.019*** | -0.005** | -0.156*** | -0.013*** |
|  | 0.002 | 0.013 | 0.002 | 0.003 | 0.015 | 0.002 | 0.002 | 0.011 | 0.001 | 0.002 | 0.018 | 0.002 |
| Mother’s age | 0.020*** | -0.080*** | -0.008*** | 0.013** | 0.006 | -0.007*** | 0.014*** | -0.041* | -0.008*** | 0.027*** | -0.107*** | -0.005*** |
|  | 0.004 | 0.021 | 0.002 | 0.005 | 0.024 | 0.003 | 0.004 | 0.021 | 0.002 | 0.005 | 0.022 | 0.002 |
| Mother’s age | -0.000*** | 0.002*** | 0.000*** | -0.000** | 0.000 | 0.000** | -0.000*** | 0.001** | 0.000*** | -0.000*** | 0.002*** | 0.000*** |
| Squared | 0.000 | 0.000 | 0.000 | 0.000 | 0.000 | 0.000 | 0.000 | 0.000 | 0.000 | 0.000 | 0.000 | 0.000 |
| Father’s age | 0.002 | -0.033*** | -0.001 | 0.006* | 0.021 | 0.004** | 0.004* | 0.014 | 0.004*** | 0.004** | 0.006 | 0.000 |
|  | 0.002 | 0.012 | 0.001 | 0.003 | 0.015 | 0.002 | 0.002 | 0.013 | 0.001 | 0.002 | 0.012 | 0.001 |
| Father’s age | -0.000 | 0.000*** | 0.000 | -0.000** | -0.000 | -0.000*** | -0.000** | -0.000 | -0.000*** | -0.000** | -0.000 | -0.000 |
| Squared | 0.000 | 0.000 | 0.000 | 0.000 | 0.000 | 0.000 | 0.000 | 0.000 | 0.000 | 0.000 | 0.000 | 0.000 |
| Notes: Robust standard errors, clustered by district, are shown in parentheses. Family size is total number of 0-20 year old children in the family at the time of the survey. Low caste is scheduled caste and scheduled tribe households, and the middle caste is other backward caste. All models include district fixed effects.  ^†^*p* ≤ .10; **p* ≤ .05; ***p* ≤ .01 | | | | | | | | | | | | |
